# The Routines-Based Model Internationally Implemented

**DOI:** 10.3390/ijerph17228308

**Published:** 2020-11-10

**Authors:** R. A. McWilliam, Tânia Boavida, Kerry Bull, Margarita Cañadas, Ai-Wen Hwang, Natalia Józefacka, Hong Huay Lim, Marisú Pedernera, Tamara Sergnese, Julia Woodward

**Affiliations:** 1 Department of Special Education and Multiple Abilities, The University of Alabama, Tuscaloosa, AL 35405, USA; 2 Department of Social and Organizational Psychology, ISCTE—University Institute of Lisbon, Avenida das Forças Armadas, 1649-026 Lisbon, Portugal; 3 Noah’s Ark Inc., Melbourne 3442, Australia; Kerry.bull@noahsarkinc.org.au; 4 Occupational Sciences, Speech Therapy, Evolutionary and Educational Psychology, Catholic University of Valencia, 46023 Valencia, Spain; margarita.canadas@ucv.es; 5 Graduate Institute of Early Intervention, Chang Gung University, Tao-Yuan City 33302, Taiwan; awhwang@mail.cgu.edu.tw; 6 Institute of Psychology, Pedagogical University in Krakow, 30-084 Kraków, Poland; nmjozefacka@gmail.com; 7 Rophi Consultancy, Singapore 329563, Singapore; honghuaylim@gmail.com; 8 Telethon Paraguay Foundation, Asunción 2420, Paraguay; marisupedernerag@gmail.com; 9 The Regional Municipality of York, Newmarket, ON L3Y 6Z1, Canada; tamara.sergnese@york.ca; 10 New Zealand Ministry of Education, 1666 Auckland, New Zealand; julia.woodward@education.govt.nz

**Keywords:** routines, intervention planning, collaborative consultation, international, implementation

## Abstract

Professionals from 10 countries are implementing practices from the Routines-Based Model, which has three main components: needs assessment and intervention planning, a consultative approach, and a method for running classrooms. Its hallmark practices are the Routines-Based Interview, support-based visits with families, and a focus on child engagement. Implementers were interested in actual practices for putting philosophy and theory into action in their systems and cultures. We describe implementation challenges and successes and conclude that (a) models have to be adaptable, (b) some principles and practices are indeed universal, (c) we can shape excellent practices for international use, and (d) leadership is vital.

## 1. Introduction

In Minga Guazú, in the hot eastern side of Paraguay, where many of the families in early intervention (birth–6 years of age) are indigenous Guaraní, a young occupational therapist (OT) welcomes a family to the early intervention center. The center is for children with physical disabilities. This OT has been trained in the Routines-Based Model (RBM) and, today, she will talk to the family about 2 or 3 of the 12 goals on the child’s and family’s intervention plan. 

Meanwhile, in Lisbon, a physical therapist (PT) is going on a home visit. The family has 10 goals, and this PT will talk to the family about, perhaps, 3 of these goals. In one of them, she will ask the family whether they would like to show her what they have been doing, and she will guide them through some strategies that, together, they have decided might help the child participate meaningfully in breakfast time.

In Cieszyn, Poland, workers are still hammering nails, as a dorm on a university campus is being remodeled to become a preschool (“kindergarten” in Polish parlance) following the Engagement Classroom Model. This model demonstration site will show how you can run a classroom to promote child engagement.

In this article, we discuss the Routines-Based Model—how it became of interest internationally, what practices implementers adopted, what challenges they faced, what successes they had, and our conclusions about what has to happen to improve early intervention around the world.

## 2. The Model

**Figure 1:**
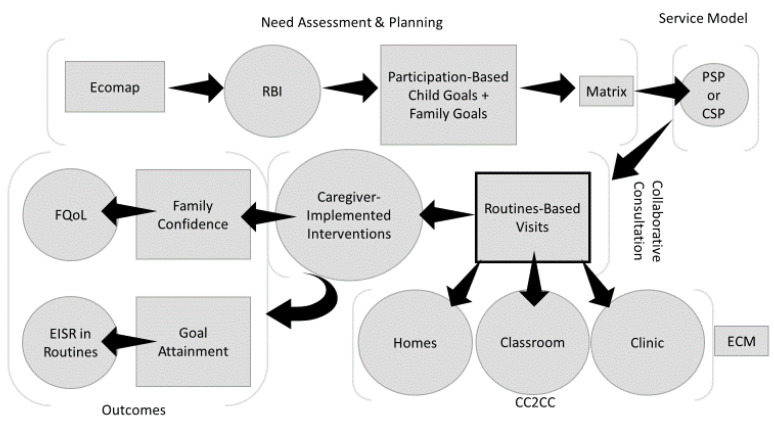
Flow of the Routines-Based Model. RBI: Routines-Based Interview, PSP: primary service provider, CSP: comprehensive service provider, FQoL: family quality of life, EISR: Engagement, Independence, and Social Relationships, ECM: Engagement Classroom Model, CC2CC: collaborative consultation to children’s classrooms.

McWilliam and colleagues developed the Routines-Based Model over many years [1,2]. The model has three main components: needs assessment and intervention planning, a consultative approach, and a method for running classrooms. The model focuses on children’s functioning in everyday routines and on family needs, strengths, and capacities. Figure 1 shows the flow of major components of the model.

### 2.1. Needs Assessment and Intervention Plan Development

In the RBM, nothing good can happen unless we have a list of goals meaningful to the family and other caregivers spending time with the child [3]. To develop an intervention plan, which goes by different names in different countries, we conduct an ecomap and a Routines-Based Interview (RBI), from which the family chooses functional goals and family goals. This RBI is the assessment of needs upon which the entire goal plan rests.

Ecomap. The ecomap is a picture of the family’s ecology [4]. The professional draws the ecomap by asking the family questions about the frequency of their contact with friends, extended family, and neighbors and how much they like the professionals and agencies they work with. Along with formal supports, it identifies the family’s informal supports, which is most important. Most early intervention services do no find out about the informal supports the family might be able to count on for support before resorting to formal supports.

Routines-Based Interview. The Routines-Based Interview (RBI) is the best known component of the RBM, but it is only one of the components [5]. An early interventionist interviews the family about the details of child and family functioning in daily routines, and the family chooses functional child goals/outcomes and family goals. In New Zealand [6], they try to avoid “interview,” because some people thought it was a formal 2-h bombardment of the family with questions [7]. From an implementation and branding perspective, however, we encourage implementers to keep using the Routines-Based Interview because of its name recognition. 

Functional goals. Goals for child functioning are written to emphasize the child’s participation in routines, such as “Jared will participate in breakfast time, hanging out time, and outside time by using single words” [8]. Furthermore, we write the goals with criteria for acquisition, generalization, and maintenance, such as “We will know he can do this when he uses five different single words, in two of these three times of day in one day, over four consecutive days.”

Family goals. As a result of the RBI, the family chooses goals for themselves and siblings of the child but not of adults not present at the RBI. The most common goal is time for oneself, such as “Diane will have two hours for herself every two weeks, for 10 consecutive weeks.”

### 2.2. Consultative Approach to Early Intervention

A principle of the RBM is that the intervention occurs between visits, so the point of visits with caregivers is to build their capacity to meet child and family needs when the professional is gone (i.e., during all the other hours of the week). 

Family consultation. Family consultation involves the professional, usually a home visitor in the U.S. and other countries, working with the family to identify (a) why a child is not doing something, (b) what might be a viable solution, and (c) whether the strategy worked. This involves the professional asking many questions to find out what has being going on so far before making a suggestion [9,10,11]. He or she also asks the family whether they would like to try it out during the session and whether they think it is feasible.

Collaborative consultation to children’s classrooms (CC2CC). Similarly, when professionals see “a child” in child care or preschool, they actually go to visit the teaching staff. Again, they jointly decide why a child is not doing something, what the strategy might be, and whether it has worked. This practice is based on seven years of research on “integrated therapy” [10].

### 2.3. Engagement Classroom Model

The RBM includes procedures for running classrooms to promote child engagement, which we have dubbed the Engagement Classroom Model [12]. Implementers focus on five components:

1. Conducting an RBI to establish functional, routines-based goals;
2. Incidental teaching to address all goals in all routines, by following the child’s lead and eliciting higher-order functioning;
3. Integrated therapy, meaning specialists work with teachers in the classroom and never pull the child out;
4. Zone defense schedule to arrange the room in zones, to organize the adults, and to decrease non-engagement time during transitions between activities;
5. Incorporating Reggio Emilia concepts to promote children’s exploration, to encourage creativity in art, and to make the environment “provocative” and beautiful.

### 2.4. How the Model Became of Interest, Internationally

The profile of implementation of the RBM, globally, is shown in Table 1. Each country has had its experiences with the exploration stage of implementation, the installation stage, the extent of implementation, systemic or cultural barriers or enhancers, and leadership.

When we talk about global implementation, we need to remember that U.S. implementation counts too. The RBM is implemented, to one extent or another, in many places in the U.S. The RBI specifically or the RBM as a whole are the most frequently cited strategies for improving federal child or family outcomes in state systemic improvement plans (SSIPs) [13]. Some states, such as Alabama, Maine, and Mississippi, have adopted the model and are at different stages of implementation. Other states—four that we know of—were strong implementers but have waned in recent years, which is a lesson in implementation, specifically sustainability. Siskin Children’s Institute, where McWilliam used to work, still demonstrates most of both the home- and community-based components of the model as well as the Engagement Classroom Model, under the leadership of Deidra Love (director of the home- and community-based early intervention program) and Julie Mickel (director of the classroom programs), respectively. Community-based components are visits to children’s child care centers or preschools. In addition, the Multnomah Early Childhood Program (MECP) in Portland, Oregon, is adopting the needs assessment, intervention planning, and home- and community-based practices in a large metropolitan environment, under the leadership of Cami Stevenson (assistant administrator). MECP is the mothership demonstration site of the model. 

Implementation data have come from leaders in each of the implementation countries. [FIRST AUTHOR] has collected data from individual states and ECTA. Key informants from U.S. states and other countries have been participants in the action research involved in this implementation science. These key informants are the authors and their colleagues. Data collection has ranged from anecdotal reports to checklist data on individual practitioners.

The first non-U.S. country to show an interest in implementation of the RBM was Portugal, because McWilliam had been working intensively with the University of Porto (under the leadership of Professor Joaquim Bairrão, psychology professor) [14,15]. Eventually, the national association for early intervention adopted the model, wrote a manual, and provided sporadic training around the country [16]. Meanwhile, students McWilliam had worked with, such as Cecília Aguiar and Tânia Boavida (faculty members at ISCTE), began their own leadership in Lisbon.

Spain followed suit, when Marga Cañadas (director of an early intervention program at the Catholic University of Valencia, UCV) became an indefatigable ambassador for the approach. Within countries, the spread of the model is something that we try to keep track of. In Spain, for example, implementation sites are gradually growing and, even within *comunidades autónomas* (states) such as Castilla-La Mancha, officials are documenting the extent to which the RBM is being adopted. Currently, Pau García Grau, also on the faculty of UCV, is training professionals there on the RBI, and Catalina Morales Murillo (on faculty at La Universidad de la Rioja) has also trained Manchegos and Manchegas in RBM practices. Some Canadians were interested, in particular Kamal Haffar, who worked to spread the word in Ontario. In Taiwan, Ai-Wen Hwang went about conducting research on the model in Taiwan, and she has produced the only randomized control trial on the RBM [17]. This study showed that the RBM (called Routines-Based Early Intervention then) group had a faster progress rate in self-care functions and independence in social functions in the first 3 months of intervention and at the 6 month follow up than the traditional home-visiting group. Traditional home visiting was not more effective on any outcomes measured.

In Singapore, Lim Hong Huay led Project ECHO, which used the model for classroom practices. In New Zealand, Julia Woodward and colleagues at the Ministry of Education decided that all children and families receiving early intervention through their system would receive practices under the RBM. In Australia, a program in Victoria and Canberra, Noah’s Ark, under the leadership of John Forster and his lieutenant, Kerry Bull, implemented the model. Meanwhile, in Western Australia, Denise Luscombe was also using many of the model’s practices in her training and consultation to others in the Perth area. Through connections Cañadas had made, a large agency serving children with physical disabilities, Teletón Paraguay, adopted the model. This agency is one of a number in Oritel, a federation of Teletones, meaning that we might have the opportunity for implementation throughout Central and South America. The most recent implementer has been an agency in Silesia, in Poland, which has constructed classrooms expressly designed to implement the Engagement Classroom Model, under the leadership of Krystian Kroczek, Lucyna Legierska, Sylwia Wrona, and, formerly, Natalia Józefacka. 

### 2.5. Why International Implementers Were Interested

International implementers were interested, first, because the model provides actual practices for implementing a family-centered approach. As professionals around the world began to hear and think about family-centered practices, they were intrigued and motivated [18,19]. However, they needed to know what to do. The perspectives and experiences reported here come from the reports of key informants—the leaders and implementers in these different countries: they are represented in the authors. For example, Portuguese early interventionists had difficulty implementing family-centered practices; they could not figure out how to implement these practices with the families they were working with. The RBM provided concrete professional practices in assessment, intervention planning, making decisions about services, and providing services in a family-centered way. Taiwanese experts were interested in the implementation stages of the RBM, which provided a guide and tools for early interventionists to follow. In Paraguay, they were interested in identifying families’ informal and formal supports, their concerns about their child’s functioning, and how to build the family’s capacity to improve child functioning. 

In New Zealand, similarly, the Ministry of Education wanted practical tools and a step-by-step process to implement family-centered principles. In particular, in New Zealand, the Ministry’s early intervention leaders wanted a way to strengthen how they worked with their indigenous Māori people to fulfill their Treaty of Waitangi obligations: to promote partnership, protection, and participation and *Tino Rangatiratanga*—Māori control over Māori affairs. They also wanted to reduce reliance on professionals, to target support at families’ day-to-day needs, to ensure everyone was clear that it is *between* visits that the intervention occurs, and to motivate adults around the child with useful intervention plans, rather than having plans sitting in filing cabinets not being implemented.

Experts in Spain saw the need for a different approach when, in 15 *comunidades autónomas*, an average of 75% of the visit (range = 51–95%) was reported to be in direct service to the child [20,21]. Spanish implementers said the model gave methods to put into everyday practice each of the DEC recommended practices [21]. This is noteworthy, because the RBM was developed before these recommended practices were chosen but perhaps it provides some informal validation for the model. 

Second, as the International Classification of Functioning, Disability, and Health (ICF) hones its methods for using subsets of the ICF for Youth and Children (ICF-CY), the RBM has proceeded to develop functional profiles [22,23,24]. In the *Measure of Engagement, Independence and Social Relationships Manual*, the alignment of ICF-CY and the Measure of Engagement, Independence, and Social Relationships (MEISR) is presented [25]. Implementers have found that the model has resulted in more professional attention to the child’s engagement, independence, and social relationships within routines—our definition of functioning. Polish implementers were attracted by the practice of following the child’s lead (i.e., incidental teaching) and the precision of the zone defense schedule, in terms of every adult in the classroom having a role at every time of day [26]. For Singaporeans, one appeal of the RBM was to move services towards a more social-inclusion approach to early intervention service delivery. Five of the 10 early childhood intervention service providers in Singapore are using selected components of the RBM. One agency has developed an implementation plan for the whole model, and another is embarking on training RBI coaches with a view to their training the staff in the RBI.

Third, many of our tools are available in languages other than English. The most common translations are into Spanish, Portuguese, traditional Chinese, and Polish. Other languages for some tools are Arabic and Slovene. Professionals have found the translations of *Routines-Based Early Intervention* [9] (Chinese, Portuguese, Korean), *Engagement of Every Child in the Preschool Classroom* [12] (Chinese), and *Working With Families of Young Children With Disabilities* [27] (Portuguese, Korean) particularly helpful.

### 2.6. Adopted Practices

What are the most commonly implemented practices from the RBM? Many implementers, such as MECP, have adopted the whole model, albeit with adaptations. In McWilliam’s experience, southern Europeans love tools. Not surprisingly, therefore, in Spain, implementers use the numerous performance-based checklists defining the major practices of the model, the MEISR [25], the Families in Natural Environments Scale of Service Evaluation (FINESSE) [28,29,30], and the Scale for Teachers’ Assessment of Routines Engagement (STARE); Casey and McWilliam, 2007). Now, owing to García-Grau’s landmark dissertation study, the Families in Early Intervention Quality of Life (FEIQoL) scale [31] is increasingly used. One characteristic of successful model adoption is when adopters consider the model their own [32]. Noah’s Ark, Teletón Paraguay, and Singaporean agencies have developed their own model, incorporating parts of the RBM that fit their cultural and organizational contexts. In Singapore, four of the organizations implementing RBM practices are part of the Thye Hua Kwan (THK) Early Intervention Program for Infants and Children (EIPIC) centers, with the others being the AWWA Ltd., Singapore. Early Intervention Centre, SPD’s (SPD was formerly known as the Society for the Physically Disabled) Building Bridges EIPIC centers, Fei Yue Community Services EIPIC centers, and Rainbow Centre Early Intervention Programme. SPD is the agency with an implementation plan, and AWWA is the one pursuing certification of coaches.

### 2.7. RBI Plus 

The RBM has a cohesive set of practices for developing a functional, family-chosen set of goals. All implementers have chosen to adopt the Routines-Based Interview (RBI), which is accompanied by an ecomap (depicting a family’s informal, intermediate, and formal supports), participation-based goals for children, and family goals for siblings and parents [33]. These three practices constitute the RBI Plus. The RBI is a needs assessment and typically results in 10–12 goals/outcomes. This long, meaty list of family-chosen goals is one of the hallmarks of the model.

Since 2009, Portugal has had legislation establishing the National Early Childhood Intervention System, but it does not define specific procedures. In 2016, the national association for early intervention produced a manual, which contained much of the RBM but also other practices, which has diluted the effect of the RBM [34]. This mixing of models is a two-edged sword: it purports to bring the best of different models together but it might diminish the effects of any one model. 

Implementers in Taiwan began with RBI Plus and moved on to the Engagement Classroom Model. New Zealand adopted the intervention planning practices and Routines-Based Home Visits and they are working towards the primary service provider (PSP) (called a keyworker in Australia and New Zealand) [35], CC2CC, and the Engagement Classroom Model (ECM). In one of the Singapore agencies, the ecomap and RBI are the most widely adopted components of the RBM.

In Australia, Noah’s Ark adopted the RBI to fully understand the family environment, conduct a functional assessment of child and family needs, and develop clear, specific, measurable goals that directly address the family’s priorities and help children develop skills relevant to everyday life. Noah’s Ark implemented the RBI by sending a staff member to be certified by Robin McWilliam, having that staff member and colleagues develop an implementation plan for 150 staff working with over 2000 families. McWilliam provided a 4 day boot camp in Melbourne to train 18 additional trainers. 

### 2.8. Family and Collaborative Consultation

A second set of practices that implementers often choose are those related to a consultative approach. In particular, York Region, in Ontario, Canada, for example, felt the RBM would assist them in moving from an expert model to a collaborative approach with caregivers, ensuring the family were the primary decision makers. International adopters of the model also implement *family consultation*, which is the collaborative-consultation method we have developed, giving families opportunity to be partners in selecting strategies for them to use with their children [36]. When professionals visit classrooms, within the RBM, they work with teachers, using collaborative consultation, rather than working directly with children. In both support-based home visits and collaborative consultation to children’s classrooms (CC2CC), caregivers might demonstrate what they or the child does, or the early interventionist might demonstrate a strategy the caregiver is interested in.

### 2.9. Engagement Classroom Model

A less commonly implemented set of practices is the Engagement Classroom Model (ECM), although the Słonezna Kraina and the University of Silesia at Katowice (Cieszyn campus) are implementing the ECM [12]. It includes the RBI, integrated therapy, incidental teaching, the zone defense schedule, and Reggio Emilia features. One of our American sites, MECP in Portland, is working with Head Start programs and other preschools where suspension and expulsion of children with disabilities occurs at a high rate. Implementation of the ECM would reduce, if not eliminate, suspension and expulsion, because the practices include ways to keep children engaged and include effective behavior management strategies for those occasions when prevention of behavior problems has not worked.

Taiwanese implementers are using goal-attainment scaling (GAS), the Teaching Styles Rating Scale (TSRS) [37,38], the STARE [30], and the Vanderbilt Ecological Congruence of Teaching Opportunities in Routines (VECTOR) [39] to determine the interaction of the STARE and VECTOR with the TSRS.

Across implementation sites, therefore, RBI Plus (the needs assessment and intervention-planning practices), our consultative-service-delivery approach, and the ECM have been implemented. This constitutes the whole model, but the whole model is not implemented by any one program. Plans are under way to add ECM to MECP in Portland, which would make that site the most comprehensive adopter of the RBM. 

## 3. Implementation Challenges

Implementation of new practices can be exciting but it involves change. We highlight here seven areas that have been the most challenging in international implementation of the RBM. A common implementation challenge, from the perspective of the purveyor, is professionals’ claiming they are implementing a model when, in fact, they are not. Their claims are usually based on an honest belief they are doing what they understand to be the practices in the model. 

### 3.1. Natural Environments

In Spain, Marga Cañadas and McWilliam tried for 4 years to persuade early intervention programs to leave their clinics (“centers”) and go into homes and communities to implement the RBM. The two main reasons for the attachment to centers were control and money. In their rooms in their centers, professionals are in control of the session. In their centers, they can see eight clients a day, whereas, visiting natural environments, they would be able to see only four or five children and families. When it was clear professionals could not be budged out of their clinics, McWilliam made a huge concession and developed procedures for routines-based clinic visits. By the same means that home visits can be performed in a clinical fashion, clinic visits can be performed in a family-centered fashion, with obvious limitations. Taiwan [40] and Paraguay have also found it difficult to use the model in natural environments. Poland would also find it difficult, but they are focusing on the Engagement Classroom Model rather than the home- and community-based practices in the model.

***What about our “churches”?*** In working on international implementation, we came across the phenomenon of the importance of the building [2]. When agencies erect or modify buildings to house their early intervention programs, they are proud of them and want to get the most out of them. You can sense an almost religious devotion to the building. McWilliam first realized this at the Asunción center of Teletón Paraguay-the flagship center. It is a beautiful, modern, spacious building, with a remarkable curved wood-slat ceiling over the therapeutic swimming pool. Why would they not use this lovely space? 

### 3.2. Primary Service Provider

A second challenge in implementing the RBM is the use of a primary service provider (PSP), rather than having a host of different providers from different disciplines working with the child [35,41]. Many early intervention cultures, worldwide, are very discipline driven, with a view that more is better. In Poland, for example, sensory integration therapists are separate from occupational therapists, and, in most other countries, psychologists play a much bigger role in early intervention than they do in the U.S. In Spain, they have psychometricians, a discipline that does not even exist in some other countries. In Paraguay, they have “early intervention professionals” who are different from teachers, therapists, and so on. So, when implementers try to organize a PSP approach, they meet much resistance. When a primary service provider cannot be used, one provider serves as the *comprehensive* service provider, attending to all child and family needs [42].

### 3.3. Relinquishing Control to Families 

A central tenet of the RBM is that families make meaningful decisions [2]. For example, they decide on the functional needs of (goals for) the child and family, they decide on what to work on between visits, and they decide on what to focus on, in each visit. For many international implementers (and American implementers), giving families this amount of control is unusual and therefore uncomfortable. The medical or psychological model, which is common across the globe, places much control in the professional’s hands.

In Singapore, a cultural challenge is that many middle-class families have a “foreign domestic helper” in the home. This young woman from Indonesia or Malaysia might care for the child for 8–14 h a day, yet she has little decision-making power. The parents participate in the needs assessment (i.e., the RBI), yet they might know little about their child’s functioning in everyday routines. Furthermore, the domestic helper would be the person caring for the child, yet she has little say in the needs or the goals. The RBM has been described as a paradigm shift in disability services in Singapore, which, before the model, had adopted the special-education and medical-therapeutic approach. The model challenges teachers and therapists to listen reflectively and to use motivational interviewing techniques. It also challenges social workers in early intervention services to improve their child development knowledge and collaborate effectively as a team with therapists and teachers. In Singapore, social workers play a prominent role, as in Europe psychologists do. In the U.S., these disciplines are less frequently represented in early intervention programs.

In Taiwan, families and professionals alike are used to professionals giving suggestions, especially on the first visit, such as a visit to the doctor. Furthermore, the time it takes to conduct an RBI is considered a barrier in Taiwan, as it is elsewhere. In some situations, professionals (and families) think professionals should provide suggestions on the first visit: in the RBM, we do not provide suggestions during the RBI or until the family has chosen goals. Recently, the Taiwan government and early intervention professionals have promoted family-centered approaches and the ECM [40].

In Paraguay, the biggest challenges have been (a) abandoning a clinical approach and moving to a family-centered approach (including a flattening of the hierarchy between professional and family) and (b) broadening professionals’ scope beyond their formal training. In many countries outside the U.S., both professionals and families expect the former to tell the latter what to do. 

### 3.4. Lack of Follow through

In implementation planning, we encourage adopters to realize that, after intensive training, they need to keep monitoring and supporting professionals. In Portugal, for example, teams were well trained initially but poorly supervised afterwards. In New Zealand, supervision and ongoing professional development varied across early intervention sites. In Australia (Noah’s Ark), the most significant challenge has been in supporting newly inducted team leaders and subsequent key workers in reaching fidelity to the model in a timely way. York Region, Ontario, listed resources required for ongoing training as a challenge.

### 3.5. Organization and Reorganization

The geographic and administrative organization of services-especially change in organization-can wreak havoc on implementation of the RBM. For example, Portuguese early intervention is overseen by three ministries, which is extremely challenging. Mississippi moved from nine regions to three. A change like that requires much energy and time, distracting from implementation. In New Zealand, the national practice support network, which had been active early in implementation, was disestablished, reducing the amount and quality of communication among districts.

### 3.6. Checklists

Implementation of the model over time requires people to observe and provide feedback—actual training and maintenance. This presents a challenge in terms of who is available and of scheduling. In Poland and New Zealand, for example, they are concerned about who will have the time to make all the checklist-based observations. Implementers, such as York Region and our Portland, OR, site, have found it difficult to find the time to ensure interobserver agreement.

### 3.7. You Are Doing It Wrong

**Figure 2:**
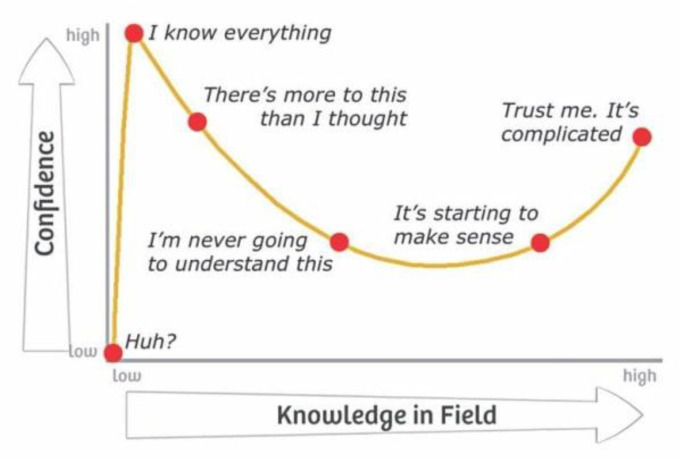
The Dunning–Kruger effect [44]. Reproduced with permission from Stanford Brown, retrieved from https://stanfordbrown.com.au/finance-101-the-dunning-kruger-effect/.

Popular on the internet are articles about what we are doing wrong, from parenting to cutting cucumbers to facing the future. With the RBM, some experts and practitioners believe that they are using the model when they are not. We see this challenge all over the place. This phenomenon is known as the Dunning–Kruger effect, where implementers with little knowledge of the details of the model have much confidence in their ability to execute the practices (see Figure 2) [43]. It is particularly difficult because the believers see no reason to change, and it is bad for the model: people see bad practices and hear they are part of the RBM. We often have to be clear about what does *not* constitute the RBM.

In Spain, we have been concerned that professionals obtain some of the increasingly available materials and tools and use them with no training. For example, professionals have been known, not only in Spain, to conduct an ecomap and then file it away, never to use it in supporting the family.

In general, change is often difficult. For some professionals, the change is welcome: they get to do what they instinctively would do or they have a structure for what they were doing. For others, it is simply a change, and accommodating to new ways of doing things is inherently difficult. For yet others, the focus on child functioning in routines and the empowerment of families is not what they believe early intervention should be about. For them, the change is the hardest. It does not help when some families want the expert approach [45].

## 4. Implementation Successes

International implementers of the RBM report successes in a number of outcomes.

### 4.1. Professionals Feeling Useful

When professionals use the RBM, they feel more useful than before they adopted the model. They feel they make a difference in the areas of child and family functioning that matter to families. Before, they were focused on what they or their assessment procedures identified as deficits in child development. With the RBM, they focus on children’s meaningful participation in everyday routines and on things that families care about. In Portugal, we actually saw improved goals, in terms of functionality and measurability, improved team functioning, and more collaborative consultation [46]. Similarly, in New Zealand and Ontario, goals became more functional and not arbitrary. In Singapore, professionals report parents’ finding goals now easier to understand, leading to better “family engagement.” Professionals also find the goals more functional and meaningful in the lives of children and families and more easily attained, which has led to greater work satisfaction.

In Taiwan, 218 professionals attended training on the RBM. The outcomes were based on McWilliam’s 12 mental shifts (www.naturalenvironemnts.blogspot.com) and 10 mistakes in early intervention [47]. Professionals with less experience shifted their mindsets more than did those with more experience (*p* < 0.05) [48]. Those who had ever attended any presentations on the RBM or who had read anything about the model were more aware of the practices at pretest (*p* < 0.05) compared to those who had not. Those who received job-embedded professional development were more likely to have shifted mindsets compared to those who received other forms of professional development (*r* = 0.56–0.82). High interrater agreement on the mental-shifts scale was reported. 

In Paraguay and Singapore, professionals report listening more to families’ concerns and needs; constructing interventions in partnership with families, building on their strengths; collaborating more with each other; seeing progress using goal-attainment scaling. In Singapore, the RBI, specifically, resulted in parent engagement and empowerment as well as professionals’ feeling empowered to understand and empathize with families. Paraguayan, Australian, and New Zealand professionals have found the model works well in aligning early intervention with indigenous ways of perceiving the family system (whānau in Māori). In Paraguay, implementers are using a smiley-face version of goal-attainment scaling with Guaraní families who do not read.

In New Zealand, the well-being of staff improved, because they did not have to have all the answers. In addition, professional support has become more purposeful and focused. Our colleagues in New Zealand also reported an outcome we have seen in different parts of the world, including the U.S.: Principles of the model have been adopted in other areas of the agency’s or district’s work.

In Poland and Singapore, they have begun to see the value of really listening to parents, rather than simply giving them instructions. Furthermore, they have seen a shift in professionals’ mentality towards supporting families rather than “repairing” children. York Region reports child care staff and families more engaged and feeling they are an integral part of the team.

### 4.2. Families Feeling Confident

Across implementation sites, families feel confident about meeting children’s and families’ needs (e.g., in Ontario and Singapore). We now have an instrument assessing this confidence. In Paraguay, families are more empowered and confident, can identify their formal and informal supports, and now have their own needs addressed, rather than professionals’ priorities for the child. In New Zealand, some families are running transition (to school) meetings, which did not happen before the RBM was implemented.

Noah’s Ark (Australia) conducted semi-structured telephone interviews with 14 families participating in the RBI bootcamp training. The families said it was cathartic, beneficial, and relaxing. They highlighted the positive impact of having a professional who was warm, caring, and personable really listen to them. Many parents indicated that developing functional goals was the most valuable part of the process. Parents commented that the two-hour interview was long, but that it went by quickly. Similar results were seen in Singapore. Not all parents were thrilled about every part of the RBI, but the interviewers were new to the RBI with fidelity, and we have not matched comments to the fidelity checks. In association with The University of Alabama, Noah’s Ark has been studying the impact of the RBI on goal functionality and found that, now, goals are largely participation based, observable, and functional.

### 4.3. Children Functioning in Routines

When professionals implement the model, children function well in routines [49]. Although routines vary somewhat across cultures, families generally want their children to participate meaningfully in the different times of day. In Portugal, professionals report that implementation has led to higher family engagement, higher implementation by families of strategies, better family well-being, families’ feeling more competent, and quicker development in children. Results from Taiwan show the ECM has positive effects on child functioning [48]. Implementers such as York Region have seen children making progress based on what is important-building on engagement, independence, and social relationships rather than developmental skills in isolation that might not promote inclusion in their community.

Implementation successes, therefore, have been that professionals feel useful, families feel confident, and children are functioning in their routines. In Singapore, as an example, implementation of the RBM has brought about a paradigm shift in early intervention towards more family centeredness and a focus on functionality. It has paved the road to actualizing developmentally appropriate practices and inclusion principles.

## 5. Conclusions

The remarkable extent of implementation of the RBM, globally but not universally, can point to four general conclusions: models must be adaptable, some principles and practices are indeed universal, some experts do think globally about practices, and leadership is needed for successful implementation. Finally, we use our experiences with global implementation of the RBM to propose framework for the relationships among evidence, public policy, and adoption of best practices.

### 5.1. Models Have to Be Adaptable

Some implementers have looked for ways to work with different cultures and countries. Baumann and colleagues [50] conducted a systematic review of the literature for four evidence-based parent-training interventions, involving 610 articles. Only eight reported a cultural adaptation, and only two tested the efficacy with rigorous research methods. In addition to needing more implementation studies, we need to include cultural adaptations, especially for models used internationally. Practices developed in one country inevitably have to be adapted for implementation in other countries. With the current rigid approach to implementation fidelity, we can expect little international replication. Seeking the balance is the key.

Obsession with evidence is an American preoccupation, and the RBM is no exception [51,52,53]. One of our tenets in the model is to eschew non-evidence-based practices, which means we have to discard bogus practices overseas (which abound) and in the U.S. [54].

However adaptable a model is to local customs and preferences, implementers might still move on to other models. Many implementers have trouble with sticking with a model over a 5 year implementation plan and having the courage to stay away from other bright shiny objects that come along and divert attention and resources from the model.

### 5.2. Some Principles and Practices Are Indeed Universal

The principles undergirding the RBM appear to be universal: we want children to function well in their natural routines, we want families to have the confidence to teach their children through their natural parenting, we want professionals to build caregivers’ capacity, and we want supervisors and trainers to use observation and feedback. Practitioners and administrators no longer want to hear about rhetoric, theory, advocacy, and esoteric research, such as “recommended practices” [21] or a “family-centered model” [55]: They want to hear about specific, evidence-based ways of doing things. 

With respect to natural environments, specifically, when professionals concentrate on *what* happens during the visit more than *where* it occurs, they can truly build the caregiver’s capacity and end up with more intervention for the child than a hands-on approach. If purveyors insist on supports being provided in natural environments, they will not be implemented at scale overseas. Portugal, however, did pass legislation and provided training, so early interventionists do make home and community visits.

### 5.3. Promoting a Global Perspective on Excellent Practices

Some American-developed models are designed to address American contexts and are difficult to implement outside the U.S. Early intervention in the U.S. can be considered somewhat ethnocentric. Many professionals assume the early intervention landscape internationally is like the U.S. one. However, U.S. perspectives can be international: for example, the headquarters of the International Society on Early Intervention is in Seattle, because that’s where its founder and president, Michael Guralnick, works. Further, the scholarly journal *Infants and Young Children* under the editorship of Mary Beth Bruder, deliberately publishes international research and perspectives and even has an international editor (self-disclosure: it is McWilliam).

Many practitioners overseas and in the U.S. are impressed with entertrainment, but we need to focus on specific practices and models. *Entertrainment* is a portmanteau of entertainment and training and refers to an appealing presentation with few practical strategies and no provision for follow-up observation and feedback (i.e., real training). Implementation can only happen if implementers commit to serious, long-term, job-embedded professional development [56].

### 5.4. Leadership

The purveyor must be in control and has to relinquish control. The purveyor (in this case, Robin McWilliam) has to be the arbiter of what constitutes fidelity to the model [57]. The purveyor has to protect and promote this fidelity for two reasons. One is that any evidence on which the model is based involved specific practices, so deviation can render the practice no longer evidence based. The second reason is a branding and credibility one: If people are saying they are using a model or its practices, when in fact they are not, any success or failure of their efforts will not really reflect the model.

Local leadership is the key [58]. In every successful implementation of the RBM, one or more local leaders have gone through the stages of implementation to explore options, decide on the RBM as a solution to needs they saw, shepherded the initial implementation, and planned for full implementation. Some of our sites are well into implementation and others are near the beginning, but the role of key players, many of whom are authors of this article, cannot be underestimated. 

Because this model was developed in the U.S., one might think the implementation over the past 15 years has been one way. Actually, as we have mentioned a number of times, to make it truly a global model, we have had to make modifications. Implementers from different countries have learned from each other. For example, Marisú Pedernera, the bright young linchpin for the Paraguayan implementation, corresponds with a colleague in Taiwan, with Cami Stevenson in Portland, Oregon, and with colleagues in Spain. Many implementers and other international professionals interested in the RBM are members of The RAM Group, a community of practice dedicated to the exchange of information about the model (www.ramgroup.info).

### 5.5. Framework Regarding Evidence, Policy, and Implementation

The role of the government, through policies or legislation, in early intervention varies greatly. In most countries, its primary concern has been the provision of services, ensuring services are available to young children with disabilities. Not all governments have been involved with the quality of services. The United States has provisions built in, such as the requirement that services be provided in natural or least restrictive environments. Different state systems of early intervention or preschool special education have varying levels of interest in the quality of services. Some, such as Alabama and Maine, have invested heavily in improving and maintaining high quality services. Outside the U.S., Portuguese law deals with the establishment of the system for managing early intervention, with a nod to quality by requiring the International Classification of Functioning, Health, and Disability be used in assessment [22,59]. In Australia, the government has established the National Disability Insurance Scheme, ostensibly to provide consumer choice, yet the scheme challenges family-centered practices and promotes a multidisciplinary approach to early childhood intervention [60]. 

**Figure 3:**
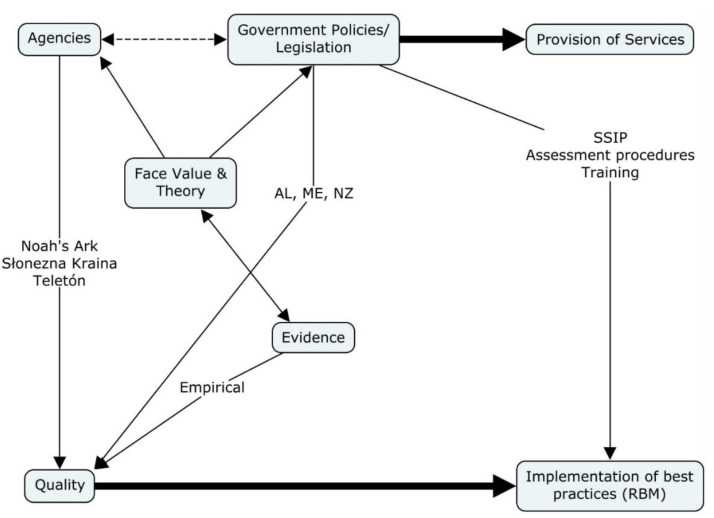
Relationships among government, evidence, and implementation. AL: Alabama: ME: Maine, NZ: New Zealand. RBM: Routines-Based Interview.

As shown in Figure 3, the provision of services comes largely from the impetus of government policies and from legislation. To some extent, we have seen that the face value of RBM practices and the theories underpinning it have had some impact on systems. Those systems concerned about the quality of services have invested in training on, for example, the RBM. New Zealand’s Ministry of Education adopted the RBI initially in one region, then spread it to the other three regions, and now uses other aspects of the RBM. They, like Australia, had a leg up because they were already using keyworkers, who are like primary service providers in the RBM. We see that the common sense of the RBM, the family-centered principles, and the theories about child functioning have had more impact on impact by agencies and governmental agencies than has the empirical evidence. However, the RBM’s face value has credibility because of its basis in evidence-based practices. 

Agencies in various international implementation sites have had a key role. To some extent, they might influence governmental agencies, and, certainly, they are influenced by the governments (i.e., they usually exist within the constraints of governmental regulations). In Australia, we have see Noah’s Ark, in Melbourne and Canberra, embrace high-quality supports to children and families-not only with the RBM but also other philosophically and empirically sound models. They have been vocal in warning the National Disability Insurance Scheme about challenges to quality. In Poland, the implementation site for the Engagement Classroom Model, Słonezna Kraina, repudiated the traditional Polish concepts of quality early intervention approaches and boldly adopted the RBM. Teletón Paraguay, similarly, broke with cultural traditions to reimagine quality, moving from a rehabilitation approach to a functional, family-centered approach. These agencies and others were influenced primarily by the logic and values of the RBM.

An interest in quality, therefore, has been the driving force behind implementation of best practices. Circling back to governmental input, we have seen that some actions by governmental agencies have enhanced implementation of the RBM. For example, in the U.S., 24% of the states have included practices from the RBM in their state systemic improvement plans, which are required, annually [61]. In these improvement plans, most often states have included the RBI in their assessment procedures. Finally, governmental agencies have invested in training in the RBM. For example, various U.S. states such as Alabama, Kentucky, Maine, Mississippi, Montana, and Nebraska have funded professional development in the RBM. Outside the U.S., governmental investment has been restricted largely to New Zealand. In Spain, some states (comunidades autónomas), such as Castilla la Mancha, have funded training in the model.

The interaction among governmental interest, research, and implementation is, therefore, a complicated and fascinating one. Pragmatists would like to find the perfect solution. Descriptivists, however, are content with admiring the phenomenon.

We end with three inferences. First, working with implementers in a caring, respectful, and honest way leads to appropriate modifications of the model. Second, sustaining an innovation is harder than implementing it: things change (funding mechanism, geographical organization, leadership), and carrying out the innovation with fidelity to the model is shaken. Third, some practices are good for young children with disabilities and their families, wherever they are. These practices might be that early intervention and parenting should help children participate in their lives meaningfully, that early intervention should support families to be the caregivers they want to be, and that early intervention professionals should know what they are doing. International implementation of the RBM has, therefore, provided a prototype for how to work across cultures, countries, and customs. 

## Figures and Tables

**Table 1:** Stages of Implementation by Locations.

Location	Exploration/Introduction	Installation/Implementation Planning	Extent of Implementation	Systemic/Cultural Barriers or Enhancers	Leadership
Australia	Presentations at national conference	Systematic planning at Melbourne/Canberra agency	1 large agency in Melbourne and Canberra; sole trainer in Perth. Limited to RBI.	National Disability Insurance Scheme poses challenges; system already implementing PSP (key worker).	Agency head and key employees in Melbourne/Canberra. Individual PT in Perth. Both closely affiliated with national professional assoc. for early childhood intervention.
Canada	Presentations at Ontario mental health/early intervention conference	Systematic planning in York Region	Full model being implemented with dedicated coach.	Separate staff for home-based services from itinerant services, with different funding sources.	Regional leaders committed resources to certification of trainers.
New Zealand	Presentations followed by more intensive training sessions, primarily on RBI	Commitment by the Ministry of Education to implement the model	Whole model implemented but lapse in fidelity measures.	Coaches initially assigned, then withdrawn, now reinstated.	Initially, one of four regions, then national leadership.
Paraguay	Spanish leader in RBM implementation introduced large rehab agency to the model	After visits from the purveyor, the agency committed to implementation and sent a staff member to study with the purveyor	Implementing the RBI, without fidelity checks, and routines-based clinic visits. Planning home visits.	Rehab center philosophy, historically. Many families are rural, extremely poor, with domestic violence.	Leaders of the agency have invested in the model. Currently, one coach carries the load.
Poland	Shared platform at international conference led to presentation at university conference	Decision to found a preschool classroom using the Engagement Classroom Model (ECM)	Classroom built to accommodate the ECM, which includes RBI.	Highly therapy-focused approach to EI 0-6. Heavy governmental involvement in curriculum.	Owner, directors, faculty member, and coach all part of tight-knit group making decisions with the purveyor
Portugal	Purveyor had been teaching classes in Porto for years Students wrote grant to study engagement	National professional organization wrote manual based largely on RBM and offered training	Whole model is endorsed, although fidelity of implementation is unknown.	Two key players in implementation have died within one year. Difficult to achieve national consensus on approach to EI. First country outside U.S. to adopt practices.	Historically, very senior faculty member, then his acolytes and their students pushed implementation. Three leaders remain who could energize implementation.
Singapore	One developmental pediatrician invited purveyor to present Other agencies became interested	Three agencies showed interest in in-depth implementation and developed separate plans	Different agencies have committed to different amounts of the model.	Service historically have been in group sessions with therapists. Caregivers are often domestic workers. Culturally, education is formal, not play based.	In each of the three major agencies, leaders have pushed for continued professional development
Spain	Purveyor asked to consult and present in Valencia and for national audience	University-affiliated EI program adopted RBI. ECM not as successful	Training now occurring in some states, primarily on RBI. Confederation of agencies endorsed the model.	Confusion between the “family-centered model” and RBM has slowed implementation. Historically, EI provided in centers.	University faculty have led the charge, establishing model demonstration projects, conducting research, providing training to master’s students, and training programs.
Taiwan	Purveyor asked to make long presentations	Core group, primarily of PTs, interested in adopting and studying practices	Interest began with routines-based visits, then Engagement Classroom Model. One model demonstration preschool opened in Taichung.	Services have traditionally been hospital based. Demonstration preschool very different from most preschools.	Taiwanese professional org. for EI involved, researcher has led the way, PT leaders critical, core group of 7 trained in the RBI.
USA	The model was developed here and has increasingly become known through workshops, presentations, and certification institutes	Implementation plans have been developed in Multnomah County, OR; Maine, Missouri, Alabama, Colorado, Montana, etc.	Currently, Maine is the flagship implementer. Multnomah County, Alabama, and Mississippi are currently being trained to implement the full model.	History of year-by-year planning for personnel development has not led to a culture of implementation. Loathing of endorsing a model has resulted in stunted practice development. Culture of going after the latest bright, shiny object has meant little multi-year commitment.	Key individuals can be identified in each of the strong implementation sites. Always, the top person needs to be on board, if not the leader. Often, someone just under the leader is the flag bearer. Those flag bearers are more successful when they have co-conspirators.

RBI: Routines-Based Interview, PSP: the primary service provider, EI: early intervention.
